# Optimizing Calcium Detection Methods in Animal Systems: A Sandbox for Synthetic Biology

**DOI:** 10.3390/biom11030343

**Published:** 2021-02-24

**Authors:** Elizabeth S. Li, Margaret S. Saha

**Affiliations:** Department of Biology, College of William and Mary, Williamsburg, VA 23185, USA; esli@email.wm.edu

**Keywords:** calcium imaging, calcium indicators, synthetic biology, GECI, calcium dyes

## Abstract

Since the 1970s, the emergence and expansion of novel methods for calcium ion (Ca^2+^) detection have found diverse applications in vitro and in vivo across a series of model animal systems. Matched with advances in fluorescence imaging techniques, the improvements in the functional range and stability of various calcium indicators have significantly enhanced more accurate study of intracellular Ca^2+^ dynamics and its effects on cell signaling, growth, differentiation, and regulation. Nonetheless, the current limitations broadly presented by organic calcium dyes, genetically encoded calcium indicators, and calcium-responsive nanoparticles suggest a potential path toward more rapid optimization by taking advantage of a synthetic biology approach. This engineering-oriented discipline applies principles of modularity and standardization to redesign and interrogate endogenous biological systems. This review will elucidate how novel synthetic biology technologies constructed for eukaryotic systems can offer a promising toolkit for interfacing with calcium signaling and overcoming barriers in order to accelerate the process of Ca^2+^ detection optimization.

## 1. Introduction

Calcium ions (Ca^2+^) are an ancient and ubiquitous second messenger with diverse functions in cells in a wide array of cell types, including muscle cells, neurons, glial cells, immune cells, and oocytes. Calcium signaling plays a key regulatory role across numerous physiological processes, including cell growth, gene regulation, neuronal synaptic activity, immune system function, hormone secretion signaling, fertilization, and the biomechanics of contraction [1]. Numerous molecular pathways, such as inositol trisphosphate, adenosine triphosphate (ATP), prostaglandin E2, and nitric oxide, mediate Ca^2+^ release [2]. Tightly controlled by numerous channels and receptors, intracellular Ca^2+^ concentrations can range from nM to µM depending on the microenvironment, from approximately 100 nM in the cytoplasm to 100–800 µM in the endoplasmic reticulum (ER) to 1–2 mM in the extracellular fluid [3,4,5]. Cytosolic Ca^2+^ signals can be classified as either transient (brief, small peaking due to Ca^2+^ influx from membrane channels), sustained (prolonged increase following influx from the extracellular matrix and internal Ca^2+^ stores), or oscillatory (repeated brief increases in free calcium), as defined by Uhlén and Fritz [6]. Ca^2+^ thus operates over a wide dynamic range to regulate physiological processes, triggering exocytosis of neurotransmitters over a timescale of a few hundred microseconds or driving gene transcription or cell division with sustained Ca^2+^ levels over minutes to hours [7,8].

Given the importance of calcium signaling in such a wide array of biological contexts, there has been an enormous amount of research devoted to unraveling the molecular players and pathways as well as the downstream effects of this activity. A crucial aspect of studying the role of intracellular Ca^2+^ in these varied processes is having robust and accurate means to detect and visualize Ca^2+^ dynamics in both in vitro and in vivo conditions. The unique spatial and temporal dynamics of free Ca^2+^ levels have necessitated high-resolution methods to facilitate detection and analysis for advancing understanding of the ubiquitous role of Ca^2+^. A successful method relies on how precisely it can bind specifically to Ca^2+^ over other ions within a proper range of concentrations, and decades have been spent focusing on developing and optimizing tools that couple fluorescent sensors with live-cell imaging.

The interconnectedness of Ca^2+^ with many molecular pathways generates complex networks within cells that establish feedback and feedforward loops, enabling multiple input signals to direct cellular level information processing, similar to modern electronic devices [9]. By using engineering principles, the goal of synthetic biology is to improve the reliability of controlling cellular behavior. User-designed inputs act together to produce a desired output in a circuit by combining standardized biological components, called “parts” [10]. These parts have been derived from natural or synthetic sources and further repurposed in recent decades to generate increasing complexity of engineered gene circuits. Synthetic biology has provided the tools needed to control and analyze endogenous biological systems, repurposing whole systems for useful functions. The majority of foundational work in synthetic biology has focused on bacteria, particularly the model organism *Escherichia coli*, because of its robust amenability to genetic engineering. On the eukaryotic end of the spectrum, synthetic biology has mostly focused on the yeast *Saccharomyces cerevisiae*, but this has brought attention to the difficulties in translating work to multicellular eukaryotes that more extensively involve RNAi, alternative splicing, and chromatin [11,12,13]. However, the recent expansion of a toolkit which can incorporate RNAi-based synthetic regulators (transcription factors, RNA-binding proteins) and engineered cell-signaling components have expanded the capacity to classify mammalian cells and regulate cell fate or morphology (see reviews [14] and [9]). These tools have current and potential applications with respect to targeting intracellular calcium signaling pathways with precise spatial and temporal control while reducing off-target effects.

Synthetic biology can thus accelerate the decades-long, ongoing process of Ca^2+^ detection optimization. This paper will review the most recent advances in Ca^2+^ detection methods to address limitations and extend the functionality of calcium-binding dyes, genetically encoded calcium indicators (GECIs), and calcium-responsive nanoparticles in a range of organisms. We go on to highlight the advantages and current limitations to establish a broad overview of the extent to which current synthetic biology tools have already been utilized to enhance study of calcium signaling and the enormous potential for synthetic biology to enhance and expand Ca^2+^ detection in animal systems even further.

## 2. Synthetic Calcium Dyes

### 2.1. Overview

The first instance of being able to monitor cellular calcium signals directly came from the introduction of organic calcium indicators by Roger Tsien’s lab in the 1970s. More generally, these indicators only bind and interact with free Ca^2+^ ions, resulting in a range of spectral changes once bound to Ca^2+^ that can be recorded by imaging. Tsien et al.’s introduction of fluorescent polycarboxylate dyes derived from the prototypical BAPTA chelator sparked rapid expansion of novel Ca^2+^ measurement techniques. In comparison to the choices of known Ca^2+^-selective chelators such as the previous nitr series, they specifically modified BAPTA due to its following important properties: over 10^5^-fold higher selectivity for Ca^2+^ over Mg^2+^ and protons, greater insensitivity to pH variations, and fast Ca^2+^-binding kinetics [15]. Specifically, they introduced indicators Fura-2 and Indo-1 and, later, visible-wavelength indicators Fluo-2 and Rhod-2, which have been widely applied in neurons and other cell types [16,17]. This section will cover the various dyes and the properties that users must consider in choosing which ones are most appropriate for experimental applications, along with the potential points where synthetic biology can enhance the optimization process for calcium dyes.

### 2.2. UV and Visible Light Dyes

Basic design of calcium probes involves covalent linkage between the binding unit with an active site for Ca^2+^ to a signaling unit which is often a fluorophore. The Ca^2+^ chelator prevents fluorescence emission of the conjugated fluorophore by photo-induced electron transfer in the absence of Ca^2+^ [18]. Currently, two approaches are applied widely to assess [Ca^2+^] with synthetic dyes. Single-wavelength indicators exhibit significant changes in fluorescence intensity when bound to Ca^2+^ without shifting excitation or emission wavelengths [19]. This enables the user to reduce the spectral overlap with other fluorophores. On the other hand, ratiometric dyes cause a shift in the peak of the excitation or emission spectrum, useful because the ratio of fluorescence emission intensities are highly reproducible functions of free cytoplasmic [Ca^2+^], at the expense of increasing the spectral range [20]. Fluorescent signals from single-wavelength and ratiometric dyes each require different standard calibration or normalization procedures which must be determined in situ.

Calibration procedures are conducted by recording fluorescent signals that correspond to precisely manipulated external [Ca^2+^]. In order to assess dye fluorescence, the addition of an ionophore, commonly ionomycin, to a dye-loaded cell allows external and internal [Ca^2+^] to reach equilibrium before measurement [20]. When absolute values are not necessary, the normalization of single-wavelength calcium dyes can be conducted by dividing the change in fluorescent signal by the average resting signal, seen as ΔF/F_0_ [19]. Ratiometric dyes enable the user to compare resting Ca^2+^ levels between experiments by dividing fluorescent signal intensity in the bound-Ca^2+^ state by free-Ca^2+^ state to derive F_Ca bound_/F_Ca free_. When absolute values are needed, performing a calibration incorporates fluorescence in the absence of Ca^2+^ (F_min_) and at saturating Ca^2+^ (F_max_), or fluorescence ratios at zero (R_min_) and saturating (R_max_) levels for ratiometric dyes. Calibration procedures for Ca^2+^ in subcellular compartments are similar (to reference specific equations used to calibrate or normalize, see Paredes et al. [19]). Peak ΔF/F_0_ values or F_max_/F_min_ values provide a useful parameter for the dynamic range of indicators (see Table 1).

The range of available ultraviolet (UV) and visible-light dyes cover a broad spectrum of affinity for Ca^2+^, enabling detection from <50 nM to >50 μM, such that high-affinity indicators (K_d_, or dissociation constant, <1 μM) quantify Ca^2+^ levels in the cytosol while low-affinity indicators (K_d_ >1 μM) detect Ca^2+^ in subcellular organelles which have higher concentrations [19]. For reference, Table 1 lists the Ca^2+^ affinities in addition to other properties of the most commonly used indicators. The earliest dyes, Fura-2 and Indo-1, enabled absolute calibration of [Ca^2+^] by measuring the ratio of fluorescence emitted at two different excitation or emission wavelengths and are still in use today to track intracellular release of Ca^2+^ in neuronal and immune cell cultures [18,32,33]. However, UV excitation precludes deep-tissue imaging since it has less penetration depth than visible wavelengths. While conventional visible-light excitation calcium indicators can improve resolution for APs in acute slices and organotypic slice cultures, they can still suffer from a low signal-to-noise ratio (SNR) in vivo, potentially due to fluorescence signal scattering by thick brain tissue [23,34]. The light scattering and aberrations as a result of refractive index inhomogeneities of tissue often limits depth penetration to a few hundred micrometers, such as the upper cortical layers of a mouse brain [34].

Compared to the UV wavelength excitation requirements of the Fura-2 family, visible-light excitation probes, such as Fluo-4 with a fluorescein fluorophore and Rhod-2 with a rhodamine fluorophore, have higher dissociation constants (see Table 1), and red-shifted calcium sensors can address the limitations of depth penetration for two-photon imaging with UV dyes. Since fluorescent imaging techniques require external illumination from the microscope for excitation, they inevitably lead to phototoxicity and potential photobleaching. Excited fluorophores can produce reactive oxygen species which react with cellular components, though this behavior is less pronounced for longer excitation wavelengths in the red-shifted or NIR region [35]. Documented effects of illumination and reactive oxygen species, such as DNA damage and cell cycle arrest (phototoxicity) or degeneration of fluorophores leading to loss of fluorescent signal (photobleaching), especially make UV excitation-based dyes or GECIs unsuitable for long-term imaging [31,35]. Although Rhod-2 is commonly used, the red-shifted fluorescent calcium indicator Cal-590 comparatively offers improved SNR and Ca^2+^ response while additionally avoiding the tendency for Rhod-2 to accumulate in the mitochondria [24]. Using Cal-590 as the calcium indicator, Birkner et al. directed ultra-short laser pulses to minimize phototoxicity, demonstrating the feasibility of deep two-photon imaging in vivo with single-cell resolution of neuronal populations in all six layers of the mouse cortex [34]. An analogous counterpart exists in Cal-520, which has advantages over visible-light dyes Fluo-4 and Oregon Green 488 BAPTA-1 (OGB-1) by improving intracellular retention and SNR. Among the dyes commonly used as fluorescent indicators for monitoring the activity of individual neurons, Tada et al. found that in vivo detection of dendritic Ca^2+^ transients with Cal-520 in Purkinje cells of anesthetized mice exhibited a high SNR and fast decay time [23].

### 2.3. Intracellular Delivery of Dyes

Calcium indicators often require invasive and time-consuming loading procedures, including diffusion from patch clamp pipettes [36], electroporation [37], microinjection [38], ballistic delivery [39], and transfer via liposomes [40]. While temperature and cell type must be taken into account, the simplest salt form of the indicator can generally enable recording within a 30 min to 1 h window. Dextran-conjugated forms of calcium dyes can be specifically utilized to address compartmentalization issues that arise when the salt forms eventually begin to clump into membrane-bound vacuoles [28]. Since both salt and dextran forms are both impermeable, readily available Ca^2+^-binding dyes can alternatively be loaded into cells as acetoxymethyl (AM) esters in a membrane-permeant, Ca^2+^-insensitive state [3]. Once inside the cytoplasm, they are cleaved in the cytoplasm by endogenous esterases, which traps the hydrophilic dye in the cell while free carboxylate groups can participate in activating the Ca^2+^-binding site of the indicator.

Due to the physical requirements of loading dyes, most of the characterization of synthetic calcium dyes has occurred in a variety of cell cultures or tissue slices, from mice [23,32,41], rat [42,43], *Drosophila* [44,45], *Xenopus* [46,47], and human lines [48,49,50]. In monitoring Ca^2+^ dynamics during neuronal activity in brain tissue slices, tissues undergoing calcium AM staining exhibited comparable functional ranges of the dye, intracellular [Ca^2+^], and viability of neurons even after incubation for >24 h within a carefully controlled recovery chamber [51]. However, the goal in intracellular studies has shifted from extending beyond cultured cells and tissue slices into organ systems in living animals. As reviewed by Russell et al., differences between brain slice preparations and in situ recording of dendritic signals or between Ca^2+^ signals in liver cell cultures as compared to perfused intact livers has served as the impetus for developing methods for discretely delivering indicators using bulk loading to image in the intact living animal [52]. Even so, soaking early *Xenopus laevis* embryos in dyes can cause dye to be trapped or bind to the vitelline envelope, generating undesired artifacts [53]. Despite ubiquitous and robust use of dyes in acutely isolated cells or in cell cultures, the preferential use of GECIs in lieu of dyes in large part is due to their easier implementation as in vivo sensors.

### 2.4. Targeting for Subcellular Localization

To expand the capacity of dyes to detect Ca^2+^ in various subcellular locations, modifications to the probe structure can modulate Ca^2+^-binding affinity for systems with lower or higher molar concentrations. For example, Mag-Fura 2 and Mag-Fluo-4 are low-affinity calcium indicators which have been designed to quantify the higher concentrations of Ca^2+^ present in the ER or the mitochondria. Based on an APTRA chelator that coordinates, or chelates, two instead of BAPTA’s one Ca^2+^, these dyes have potential crosstalk interference with Mg^2+^ due to a difference in ion selectivity (see Table 1 for Mg^2+^ K_d_ values) [18]. Buffering effects for calcium probes depend on the [Ca^2+^] in the organelle, the K_d_ of the probe, and the concentration of the probe itself [54]. Particularly for cytosolic Ca^2+^ detection, the required probe concentration is often similar to the nanomolar range of cytosolic [Ca^2+^]. For example, as reviewed by McMahon and Jackson, high concentrations of Fura-2 loaded into cells can bind 99.9% of Ca^2+^ and leave the small percentage remaining as free ion [55]. While endogenous buffers (i.e., Ca^2+^-binding proteins and metabolites) have an inherent biological effect, the calcium sensor buffering is an artifact. When the buffering capacity of calcium sensors exceed endogenous buffering effects, the probe will dominate and thus alter Ca^2+^ dynamics. However, the ER [Ca^2+^] is in the micro to millimolar range, and lower concentrations of probes are sufficient for organelle Ca^2+^ detection without interfering with endogenous buffering [54].

Even so, since these synthetic dyes are not protein-based ion indicators, their targetability remains poor [56]. To improve targeting of low-affinity calcium indicators to the ER lumen, targeted-esterase induced dye loading (TED) can be utilized in vitro in cell lines, neurons, and glial cells [3,18]. A vector containing a carboxylesterase targeted to the ER with a signal peptide is overexpressed to ensure that cleavage of the AM-ester form of low-affinity dyes traps them in the ER lumen at a high concentration. Poletto et al. visualized Ca^2+^ distribution in the ER of HeLa cells by photoconverting fluorochromes using diaminobenzidine photo-oxidation. This efficiently localizes Ca^2+^ at the electron microscopy level, thus combining the specificity of available fluorophores such as Mag-Fura-2 to the high spatial resolution of transmission electron microscopy [48].

Synthetic biology can potentially improve a mechanism such as TED by using an orthogonal split esterase. The principle of orthogonality is to introduce genetic circuits that aim for maximal reduction in components cross inducible with endogenous cellular pathways. Along these lines, synthetic biology techniques may be well suited for engineering an orthogonal enzyme–substrate pair which can unmask small molecules in live cells, thereby generating both imaging agents and bioactive molecules [57]. This was recently demonstrated by Jones et al., who developed a split microbial BS2 esterase system which could detect multiple protein–protein interactions and drive cell fate changes, an increasingly important functionality of engineered gene circuits. The interaction-dependent unmasking of α-cyclopropyl esters by split esterase fragments enabled detection of numerous protein–protein interactions in mammalian cells, not limited to leucine zipper peptides and small molecule-induced dimerization domains. By continuing to explore split protein engineering and consequent output of fluorescent or chemiluminescent signal generation, we can improve analysis of calcium signaling via increased targetability and functionality of our current synthetic dyes.

**Figure 1:**
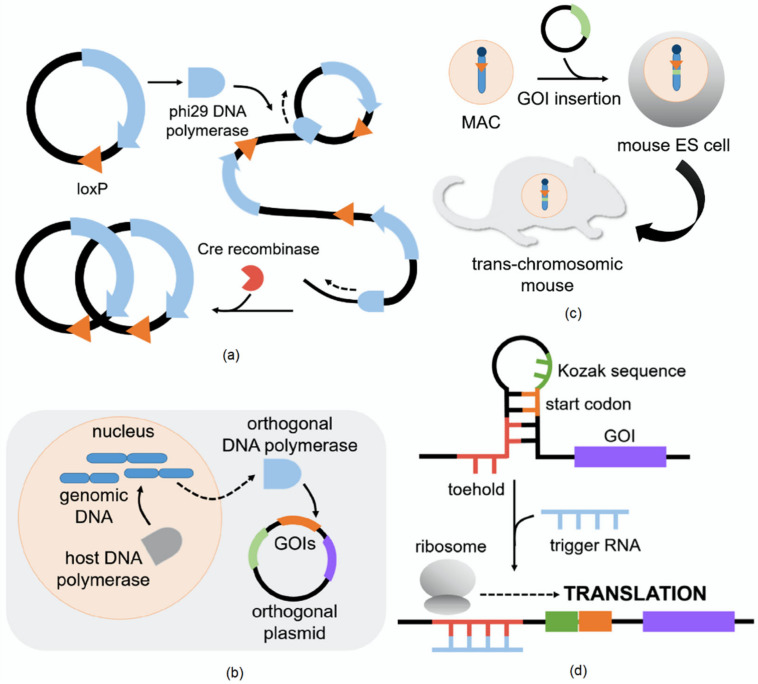
Synthetic biology techniques to improve orthogonality of expression vectors introduced into animal systems. (**a**) Minimal TTcDR [59]. A plasmid which encodes its own DNA polymerase, phi29, replicates via rolling circle replication. Cre recombinase cuts at the loxP sites, which results in recircularization of the DNA in vitro. (**b**) OrthoRep system in yeast [60]. Native machinery from yeast generates an orthogonal DNA polymerase that solely replicates the desired plasmid. This separates the replication of the genes of interest (GOIs) in the cytosol from nuclear replication of endogenous genes. While this has not yet been tested in animal systems, the yeast system acts as a proof of concept for compartmentalization of DNA replication between the nucleus and cytoplasm in eukaryotes. (**c**) Mouse artificial chromosome (MAC) stably expressed in mouse embryonic stem (ES) cells [61]. Takiguchi et al. constructed the MAC vector from a truncated native mouse chromosome. The incorporation of a Cre-loxP-mediated platform enables insertions of a GOI, and the modified MAC can be transferred into mouse ES cells to form trans-chromosomic mice. (**d**) Mammalian toehold switch for a post-transcriptional circuit [62]. Dependent on RNA–RNA interactions, it contains a toehold sequence complementary to a trigger RNA, a stem–loop structure that includes a Kozak sequence, and a start codon upstream of the gene of interest. The translation of the gene is constitutively repressed. Once a trigger RNA binds to the toehold sequence, the stem–loop structure is disrupted and exposes the Kozak sequence and start codon in order to initiate translation.

However, techniques that introduce orthogonal enzyme–substrate pairs would require the use of expression vectors, such as the lentiviral vector used to introduce carboxyesterases into cell lines for TED [3]. As reviewed by Nora et al., researchers who take on the optimization of expression vectors, especially in generating reporter cell lines, will need to consider broad-host-range vectors to allow users to easily switch between host types without requiring reconstruction of the circuit of interest [58]. While an incredibly vast array of expression vectors has been developed and optimized over the decades, synthetic biology has taken a more focused approach to ensuring orthogonality during the construction and tuning of expression vectors. This has been more successfully addressed in microbial systems, but advances in synthetic biology already offer a few exciting avenues in cell-free, yeast, and mammalian systems. Minimal transcription- and translation-coupled DNA replication (TTcDR) [59], an orthogonal replication (orthoRep) system [60], or mammalian artificial chromosomes [61] (see Figure 1a–c) present examples in more complex animal systems that have specifically undergone rigorous characterization in vitro or in vivo to evaluate and prevent crosstalk of expression vectors. These relatively recent advancements point toward future applications which may directly apply the principles of orthogonal vector expression in relation to Ca^2+^ dye targeting or discussion of GECI expression in later sections of this review.

Introducing expression vectors in animal systems that coordinate with synthetic calcium dyes should take into account the potential benefits of designing a post-transcriptional circuit. These circuits are especially useful because they often do not require nuclear entry, thus avoiding the questions of genomic integration into the host genome or epigenetic modification following introduction to the host cell line (see review [63] for a more extensive look at currently available post-transcriptional circuits). Recently, Wang et al. reported the establishment of a toehold switch system in mammalian cells (see Figure 1c) [62]. In the presence of the trigger RNA, in this case microRNAs (miRNAs) endogenously or exogenously expressed in several mammalian cell types, translation was turned on for a reporter fluorescent protein (FP). With more optimization, this strategy can be potentially applied in a circuit to produce an orthogonal enzyme–substrate pair. Post-transcriptional circuits are especially suited to detecting intracellular events, such as the loading of synthetic calcium dyes, that would need a rapid response of protein production for targeting to the desired subcellular compartment [63].

### 2.5. Multiplexing Ca^2+^ Imaging with Dyes

Multiple dyes can be combined with each other in various cell types, ranging from immune cells to cardiomyocytes to neurons. An example of a combinatorial implementation was the detection of Ca^2+^ flux by simultaneously measuring Fura Red AM and Fluo-4 AM in individual T cells [41]. Christo et al. used the ratio between the two to detect oscillations in Ca^2+^ concentration as a function of fluorescence intensity. This enabled tracking and activation of T cells on the individual level to conduct real-time analysis of the magnitude and temporal flux of intracellular Ca^2+^ release. Another study by Lee et al. demonstrated the feasibility of using red-shifted Rhod-2 AM with a near-infrared (NIR) voltage-sensitive dye, both compatible in blood-perfused tissue, to conduct multiparametric imaging of the mammalian heart for cardiac optical mapping [64]. Both examples utilize distinctive properties of a wide spectrum of available dyes to achieve even more output that can be captured with optical techniques (refer to Table 2).

One of the barriers that largely hampers more extensive use of multiplexed Ca^2+^ imaging with synthetic calcium dyes is that the majority of dyes exist only in the UV and visible-light region. In lieu of calcium indicators with emission wavelengths in the visible region, NIR fluorophores can be used as imaging contrast agents because they can suppress interference from cellular autofluorescence and light scattering while enabling multicolor imaging [4,92]. Varying levels of intrinsic biomolecules, such as aromatic amino acids, nicotinamide adenine dinucleotide, and flavoproteins, with their distinct absorption and emission wavelengths can emit different levels of autofluorescence depending on the tissue type. While significant autofluorescence comes from emission in the green and yellow regions, it is minimal in the red to NIR region. [93]. In comparison to visible-light emitting dyes, NIR dyes have longer emission lengths of approximately 650–900 nm; since tissue and water have a low background window, this allows excitation light to penetrate more deeply into the tissue and detection of fluorophore emission with better resolution in cells and tissues [4,94]. However, low absorption coefficients can lead to low brightness, and low photostability results in short bleaching time [92]. Egawa et al. had in part addressed these concerns by utilizing a far-red to NIR fluorophore, Si-rhodamine (SiR), to develop a novel Ca^2+^ probe capable of visualizing spontaneous APs in mouse cortical neurons and hippocampal CA1 pyramidal cells [29]. In brain slices loaded with a red sulforhodamine 101 specific for astrocytes and green FP in some neurons, the fluorescence wavelength region of CaSiR-1 provides a color window for multicolor imaging of Ca^2+^ APs.

As fluorophores continue to undergo future optimization, a synthetic biology approach can potentially utilize an “integration” strategy for the rational design of small-molecule, optically tunable fluorophores [94]. For example, a way to accelerate the development of NIRs in comparison to more traditionally used synthetic dyes would be to strategically install a built-in, optically tunable group into the traditional NIR fluorescent dye which can enable direct tuning of optical properties. By taking advantage of the optically tunable mechanisms from visible light-emissive dyes, researchers such as Collot et al. have created a ratiometric NIR probe [30]. A rational design strategy can facilitate further avenues of functionalization, such as coupling hydrophilic compounds such as dextrans to those calcium sensors [28]. While dextrans already exist for the more commonly used UV and visible-light dyes, the current literature has not yet incorporated NIR dyes which utilize dextran conjugation. In the future, more widespread applications of dual-imaging necessitate NIR dyes that do not have the same limitations which already have been obviated in UV and visible-light dyes over many years of optimization. This synthetic biology approach can more rapidly expand the toolkit of available dyes, taking advantage of the existing background literature on the history of optimizing previous organic Ca^2+^ dyes to inform the available optically tunable groups which can tackle issues of diffusion rates, leakage through cell membranes, and intracellular compartmentalization.

## 3. Genetically Encoded Calcium Indicators

### 3.1. Overview

**Figure 2:**
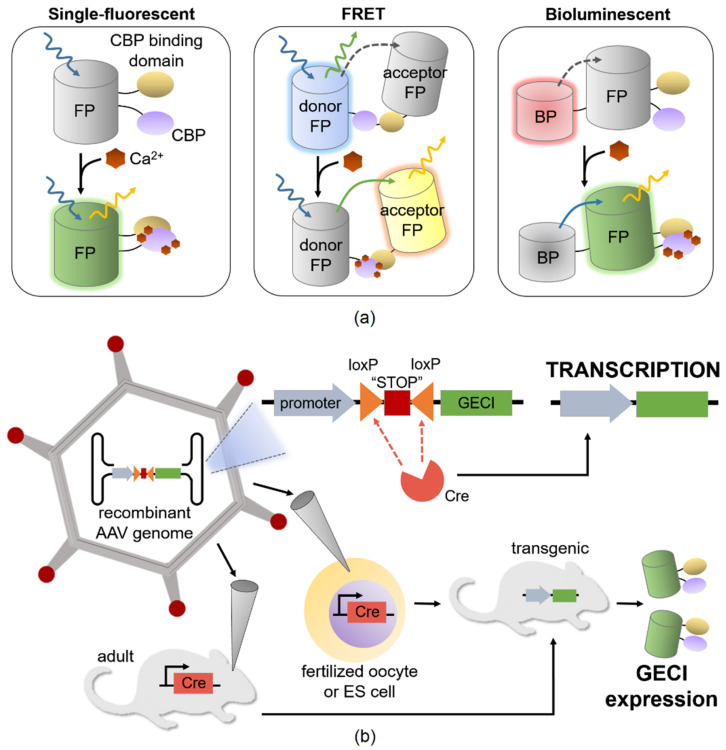
GECI Ca^2+^-binding mechanism and delivery in vivo. (**a**) A single-FP type GECI contains a calcium-binding protein (CBP) which interacts with a CBP-binding domain when bound to Ca^2+^ [98]. When the FP is excited, Ca^2+^ binding to the CBP will cause conformational changes that increase emitted fluorescence. Forster resonance energy transfer (FRET)-type GECIs include both a donor and acceptor FP [81]. When binding calcium, FRET decreases emission of the donor FP and increases emission of the acceptor FP. The bioluminescent GECIs do not require external excitation, but binding calcium enables bioluminescent resonance energy transfer and consequent emission of light from the FP [99]. (**b**) A recombinant adenovirus-associated virus (AAV) genome is engineered to contain the GECI of choice. GECI expression is inhibited by a transcriptional insulator, or a “STOP” sequence, which is flanked by loxP sites. In the most basic Cre/loxP expression system, the Cre recombinase enzyme will target the loxP sites and remove the sequences in between. Adult mice or fertilized oocytes are injected with recombinant AAV particles to integrate into the genome. For Cre-expressing cells, the “STOP” insulator is excised so that GECI expression occurs in the targeted cell type [100].

A GECI is comprised of natural protein or peptide sequences, containing (1) at least one Ca^2+^-responsive element that changes the optical property of the protein upon binding Ca^2+^ and (2) a light-emitting protein [95]. GECIs are produced by translation of a nucleic acid sequence, so the expression of indicators does not require exogenously added co-factors or chemicals. The first protein-based calcium indicator was bioluminescent photoprotein aequorin isolated from *Aequoria* jellyfish in the early 70s [96]. As fluorescence imaging achieved rapid progress in recent few decades for visualizing Ca^2+^ from the level of subcellular compartments to single cells to cell populations, GECIs have continued to grow in popularity, especially since the introduction of the early single-FP variant developed by Imoto and colleagues [97]. For a comparison of the generalized mechanisms of binding Ca^2+^ between the three classes of GECIs, refer to Figure 2a.

GECIs obviate exogenous loading of indicators and indiscriminate uptake of membrane-permanent dye esters of synthetic dyes, clear benefits which have promulgated significant optimization efforts [24]. In addition, by integrating GECIs into the genomes of transgenic organisms, they can be continuously synthesized by the host cell machinery and thus reduce the temporal limitations of dyes rapidly being cleared from the cytosol [20,101]. This allows measurement of activity in a large population of neurons and small neuronal compartments over a timeframe of milliseconds to months [66]. Over the course of this section, we will cover recent improvements and applications of the three main classes of GECIs—single-FP, FRET, and bioluminescent—and the ways that synthetic biology tools might improve the genetic delivery of GECIs into animal systems and offer high-throughput methods of detecting and optimizing GECIs.

### 3.2. Single-Protein GECIs

The most commonly used GECIs are fluorescent protein based, including resonance energy transfer (RET) types and single-FP types recognizable by the extensively utilized GCaMP variants. GCaMP is composed of a circularly permuted GFP, a Ca^2+^-binding protein called calmodulin (CaM), and a CaM-binding domain (such as the RS20 from smooth muscle myosin light chain kinase in an early GCaMP3 variant) [20]. The most recent GCaMP6 iteration uses the M13 motif from myosin light chain kinase for the CaM-binding domain [98]. The iterative process of mutagenesis has enabled improvements in brightness, SNR, and photostability over multiple generations of GCaMP variants [98,102,103]. These considerations must be taken into account in order to gain traction in research. The more advanced sensor GCaMP6 compared to GCaMP3 has resulted in a shift in use for studying Ca^2+^ dynamics in astrocytes [71,80,104] and other neuronal cells in zebrafish larvae [105] or *C. elegans* [106]. Even though a GCaMP8 variant was also obtained, it showed a lower sensitivity and baseline fluorescence in comparison to GCaMP6 in a cellular environment and has hence seen little to no use while GCaMP6 continued to gain popularity [107].

Even while optimization of the linker region and the circularly permutated GFP domain has resulted in enhanced fluorescence readout, the previously mentioned developments lacked insight into or did not improve GCaMP kinetics. Evaluated in cultured neurons, GCaMP 6s and 6m variants exhibited even larger improvements in response amplitudes, but this was at the cost of kinetics (see Table 3) [98]. Variants of GCaMP based on rise and delay kinetics—GCaMP 6s (slow), 6m (medium), and 6f (fast)—typically have a high affinity, with K_d_ values between 100 and 300 nM [108,109], which results in slow Ca^2+^ dissociation rates and signal decay occurring at 1–5 s^−1^ at 20 °C [110]. This has acted as a constraint against rapid tracking processes in excitable cells, posing a difficulty for monitoring rapid Ca^2+^ transients that require millisecond resolution and fluorescence decay and rise rates up to 1000 s^−1^ [103]. Studies that focus specifically on GCaMP response patterns in distinct synapse types from in situ imaging reveal variation in tracking efficacy [87]. Although GCaMP-s were not effective for tracking rapid dynamics of Ca^2+^ influx triggering transmitter release, they could better reflect residual cytosolic Ca^2+^ accumulation governing synaptic plasticity. The current range of GCaMP6 variants suffice for certain applications, such as the pan-neuronal expression of GCaMP 6m for whole-brain Ca^2+^ imaging in *Drosophila* [111]. Cardenas-Diaz et al. were able to characterize candidate genes in vitro in the human EndoC-βH1 cell line by generating a dual reporter to express both an insulin–luciferase fusion protein and a GCaMP6s variant [112]. The slow kinetics, wide dynamic range, and high baseline brightness of GCaMP6s was suitable for studying Ca^2+^ flux in β cells with fluorescence microscopy or pan-neuronal expression in mice and zebrafish [86,113].

The most recent decade of research has dedicated further efforts to address onset kinetics and decay rates [108,124,125,126]. As new generations of single-FP GECIs are developed, it is also necessary to conduct comparisons of spatiotemporal Ca^2+^ findings in cell processes between older and new variants. For example, Ye et al. found that Ca^2+^ waves in processes along with microdomain Ca^2+^ transients were more readily detectable using GCaMP6f instead of GCaMP3, with only minor differences in kinetics and a similar dynamic sensing range [104]. The need to detect rapid Ca^2+^ transients, such as those found in neonatal cardiac myocytes, has led to interest in so-called ultrafast variants of GCaMP6s and GCaMP6f. This has led to rational design of specific residues in the binding interface [108]. The improved calcium indicators revealed a 4-fold faster decay of ATP-induced intracellular Ca^2+^ transients in vitro compared to standard GCaMP6f. Stimulating hippocampal CA1 pyramidal neurons consequently revealed faster rise and decay times, indicating that high-frequency AP tracking may be limited by the response kinetics of calcium indicators. Specific applications that require faster kinetics to resolve spike rates in neurons, such as parvalbumin-positive interneurons, are difficult to estimate in vivo. Therefore, Inoue et al. rationally engineered an array of multicolor calcium indicators by polymerase chain reaction (PCR) mutagenesis of the Ca^2+^/CaM-sensing domain and linker sequences in order to optimize kinetics and brightness [127].

These targeted mutagenesis methods for evolutionary engineering will continue to be elevated by synthetic biology advances in both prokaryotes and eukaryotes, such as targeting activation-induced cytidine deaminases (AID) activity in yeast and mammalian cells [128] or base-editor mutators [129]. The clustered regularly interspaced short palindromic repeats (CRISPR)/Cas (CRISPR-associated) system, which will be discussed more extensively in Section 5.3 on modifying Ca^2+^-related gene elements, can form single-stranded DNA. By generating a single-stranded break, the Cas9 nickase offers a suitable substrate for AID activity to lead to mutagenesis. This synthetic complex combines the genome targeting capability of Cas9 nickases and the somatic hypermutation capability of cytidine deaminases (CD) with off-target effects comparable to those of conventional CRISPR/Cas9 systems [128]. The alternate system, EvolvR, also uses CRISPR-guided nickases but introduces an error-prone DNA polymerase variant that can be selected based on different misincorporation rates, number of nucleotides incorporated per binding event, and nucleotide bias during incorporation [129]. EvolvR can diversify nucleotides in editing windows up to 350 nucleotides in length and at a targeted mutation rate more than 7 million-fold greater than in wild-type *E. coli.* In order to reap the demonstrated benefits of the EvolvR system for rationally engineering the user-defined editing windows for GCaMP-s, an analogous method should be developed in commonly used in vitro cell lines, such as HEK293, for conducting the necessary assays to extend this prokaryotic synthetic biology tool to applications in eukaryotic systems.

### 3.3. FRET-based GECIs

Since the peak fluorescence intensity varies depending on GCaMP expression levels and cytosolic Ca^2+^ fluctuations change fluorescent intensity without an accompanying spectral shift, GCaMP variants are not suitable for quantitative ratiometric measurements despite having greater spatial and dynamic ranges in comparison to the ratiometric dyes [20,24]. Attempts at earlier “ratiometric” versions of the single-FP variety, GEX-GECO-1 and GEM-GECO1, required UV excitation, which came with tissue penetration depth and phototoxicity issues similar to those encountered by Fura-2 and Indo-1. The fluorescent FRET-based GECIs can offer ratiometric capabilities using visible light readout.

However, FRET-based ratiometric GECIs have drawbacks because of a lower SNR and slower on/off-response kinetics compared to GCaMP, in addition to necessitating extensive post-data processing [20]. To address this, Cho et al. engineered GCaMP-R, a combination of GCaMP and Ca^2+^-independent mCherry connected by an anti-FRET ER/K alpha-helix. The anti-FRET linkage between the dual fluorescent protein reporters improves SNR by increasing GCaMP fluorescence above the background. This ratiometric GECI does not fully address negative side effects that can arise from overexpression, including GCaMP6 influence on total Ca^2+^ buffering capacity or potential aggregation of mCherry in rod photoreceptors. Within the FRET-based yellow chameleon (YC) family of calcium indicators that leverage Ca^2+^ dependence of CaM binding to the M13 peptide, newer versions such as YC3.6 now offer a larger dynamic range and improved SNR [81]. The array of Ca^2+^ affinities and dynamic ranges in the YC family are suited for specific tissue type applications, in this case YC-Nano3GS detecting both basal and transient increases in Ca^2+^ in the *Xenopus* leading edge mesoderm [85].

Another means of countering these challenges is the use of a troponin C-based FRET sensor, or Twitch variant [92]. Exhibiting a linear response, small size, a wider range of Ca^2+^ affinity, better photostability, larger dynamic range, and faster kinetics, Twitch sensor variants were tested in the brain and lymph nodes of mice. Their sensitivity was comparable to that of YC3.6 and synthetic calcium dyes for applications in neuroscience and immunology, marked by visualizing APs in neurons and high-resolution tracking of T lymphocytes within an inhomogeneous tissue environment. This method is especially appealing for orthogonality in order to avoid any uncontrolled GECI interaction with endogenous proteins. While CaM has various downstream targets, troponin C has a limited interaction that includes troponin I and troponin T [130]. Interestingly, even though the in vitro dynamic range of an earlier indicator incorporating troponin C was small, it demonstrated acceptable in vivo performance that could suggest reduced endogenous interference [120]. The elaboration of orthogonality may be appealing to future synthetic biologists who must take into account off-target effects that may necessarily occur in the more common GCaMP family due to trans-activation of CaM targets.

This orthogonality should be taken a step further, re-emphasizing the importance of using approaches that can extend in vitro analysis of amplitude and kinetics of intracellular Ca^2+^ signals to in vivo studies, despite the technical challenges that present in gathering in vivo measurements [131]. Dong et al. fused together GCaMP6f and tdTomato in order to fashion a genetically encoded ratiometric calcium sensor, termed Salsa6f [72]. They first validated the protein engineering results in vitro with transiently transfected human primary T cells and then tested in vivo with transgenic mice homozygously expressing Salsa6f in CD4+ T cells (see Section 3.5 on transgenic GECI expression for synthetic biology applications). The Thestrup et al. study mentioned above also validated the in vivo efficacy of the Twitch sensor variant by observing the brain and lymph nodes of mice [92]. Kyratsous et al. expanded on these in vivo studies, using the Twitch Ca^2+^ sensor alongside nuclear factor of activated T cells (NFAT) as reporters of T-cell activation and injecting in vitro activated T cells into rats [132]. Even so, the limited optic penetration of two-photon microscopy is insufficient to screen the entire spinal cord parenchyma, which also necessitates studying Ca^2+^ responses ex vivo with acute spinal cord explants. Borgne et al. first studied naive T cell culture using mCameleon, a FRET-based GECI based on a modified version of Cameleon with a brighter acceptor fluorophore (YPet instead of YFP) [133]. Knock-in mice containing mCameleon could be utilized to observe distinct Ca^2+^ patterns in naive T cells. In the above studies, validation that genetic manipulation of the cells in vitro does not alter their normal behavior precedes measuring Ca^2+^ flux in vitro, in vivo, and ex vivo.

### 3.4. Bioluminescent GECIs

It has been well documented that FP-based GECIs have high spatiotemporal resolution but can only tolerate short-term imaging due to external illumination [92,101]. This can result in undesirable phototoxicity and autofluorescence, which has recently encouraged separate investigation into bioluminescence protein (BP)-based GECIs. An initial drawback for practical imaging was weak signal intensity. Concurrent with recent optimization of CaMP-based calcium indicator brightness, the discovery of bioluminescent proteins with improved brightness, specifically NanoLuc (Nluc) from the deep-sea shrimp *Oplophorus gracilirostris*, has narrowed the disparity in imaging quality compared with fluorescent proteins [101,134].

As of now, few studies have developed BP-based GECIs, and the optimization remains limited. Qian et al. constructed a Ca^2+^ sensor fusing Nluc to a topologically altered GCaMP6s [99]. They placed LUCI-GECO1 expression in neurons under control of an hSyn promoter, which could be easily detected by an electron-multiplying CCD camera. This served as a sensitive ratiometric GECI which still retained properties of GCaMP6s. In another study, Farhana et al. combined a single FP-based GECI with a split luciferase for a Green Luminescent Indicator for Calcium Observation (GLICO) [101]. This combination provided 2200% of the dynamic range compared to all bioluminescent GECIs, enabling prolonged observation of bioluminescence in HeLa cells, rat pituitary tumor (GH3) cells, and dissociated rat hippocampal neurons where excitation light would lead to high background signals.

The concept of bioluminescent-based Ca^2+^ imaging can be especially useful as a highly compatible strategy to complement optogenetics. Since BP-based GECIs do not require light excitation, observations of Ca^2+^ signaling can avoid functional crosstalk with optogenetic actuators. Since optogenetic methods are becoming crucial for studying a variety of spatiotemporal properties in signaling networks, they are uniquely positioned to control or modulate time-encoded inputs on cellular targets [135]. Advances such as the establishment of in situ switching of optogenetic actuators based on common blue-light LOV2 photoreceptor rely on engineered resonance energy transfer between two fluorescent proteins [136]. Given the usefulness of optogenetics in addressing fundamental questions about cellular physiology that also factor in calcium signaling, it would be extremely useful to open the possibility of using the most optimized optogenetic tools for one signaling pathway in concert with a Ca^2+^ detection method that uses a bioluminescent readout free of crosstalk.

### 3.5. Stability of GECI Delivery in Transgenics

One of the exclusive benefits to GECIs in comparison to synthetic calcium dyes is the ability to use host machinery to produce the calcium indicator, rather than a more labor intensive dye-loading process prior to imaging. Common methods of introducing GECIs include microinjection of mRNA encoding fluorescent calcium reporters at the 2-cell or 4-cell stage for *Xenopus* embryos [53,85] or via injection with adeno-associated virus (AAV) in rats and mice [137,138]. Unfortunately, some concerns can arise from long-term expression. For mice, greater than three weeks post-AAV injection resulted in toxicity in neurons, so researchers selected a two-week period to achieve GECI expression [139]. On the other hand, the *huc*-driven neuronal expression of GCaMP3 in zebrafish decreases 6–7 days post-fertilization, posing a temporal barrier for studying the optic tectum beyond those in young larvae [140]. An alternative method for achieving long-term GECI expression is to develop transgenic animal lines. For example, by generating a transgenic line expressing GCaMP3 under the control of a neural beta-tubulin promoter rather than the commonly used *huc*-driven lines, Bergmann et al. were able to extend imaging to much later-stage zebrafish.

Though GCaMP variants have wide-ranging applications, particularly for use in transgenic animals, studies in practice have documented chronic cell damages induced by GCaMP. Similar to exogenous CaM overexpression, GCaMP expression has the potential to impair health of tissues or cells. Firstly, GCaMP can disrupt Ca^2+^ homeostasis in cells, particularly because of these indicators’ ability to bind Ca^2+^ at physiologically relevant concentrations [1,89,109]. This can alter synaptic transmission, as shown by decreased synaptic vesicle exocytosis following approximately three weeks of GCaMP6m expression [109]. In addition, researchers have identified cardiomegaly and hypertrophy in the hearts of GCaMP2 transgenic mice, cytotoxicity and death in neurons, or abnormal cortical activity in multiple lines of GCaMP6 mice [115,141]. Overexpression of GCaMP can interfere with the cell buffering capacity of Ca^2+^, modify dynamic Ca^2+^ signals, fill the nucleus with GCaMP, and result in cell death [76]. High-affinity indicators can buffer Ca^2+^ transients and exhibit a slower rise and decay. Though any calcium probe exhibits buffering capacity due to its ability to bind Ca^2+^, the effect varies depending on the probe type. For example, aequorin has three high-affinity sites to bind Ca^2+^ in comparison to the four sites for GCaMP-s, but due to a high SNR, reliable aequorin measurements necessitate only moderate levels of probe expression. This consequently reduces the intracellular Ca^2+^ buffering effect, which is discussed more extensively in Section 2.4 regarding fluorescent dyes as exogenous buffers [142]. On the flip side, insufficient quantities of the sensor give a weak readout with low SNR and loss of sensitivity. Nevertheless, noticeable improvements have been made to GCaMP6 in order to address some of the pitfalls of these GECIs. Specifically, GCaMP has an unintended side effect of interfering with the gating and signaling of L-type calcium channels (Ca_V_1) because of its impaired apoCaM, which is critical to Ca_V_1 functioning [115]. This can lead to a disruption of Ca^2+^ dynamics and gene expression, facilitating detrimental nuclear accumulation as a result of acute and chronic Ca^2+^ dysregulation by interference with CaM translocation and phosphorylation of CREB. Yang et al. were able to overcome these perturbations in gating and signaling of calcium channels by incorporating an additional apoCaM-binding motif.

Even though the gene expression aspect of GECI is one of its strengths as a calcium indicator, the ramifications of longer cultivation time and phenotypic heterogeneity of transgenic cell clones can present a bottleneck to optimization of recombinant protein production in animal cell systems, as reviewed by Wang et al. [143]. The background literature on bioengineering has long recognized how the components in expression vectors, including promoters, enhancers, and additional cis-elements, influence the stability and magnitude of transgene expression [144,145,146]. For example, when studying the stability of transgene expression in an episomal vector, researchers discovered a robust regulatory sequence EF-1alpha that contributed to high-level transgene expression in transfected CHO-K1 cells [147]. More directly related to GECI expression is the recent advances in recombinant AAVs as a platform for in vivo delivery (see Figure 2b). Inserting scaffold/matrix attachment region (S/MAR) sequences into constructs is a strategy to assist episomal forms in undergoing replication in transduced cells [148]. S/MAR is a chromosomal retention element that binds nuclear matrix proteins to provide stability for the episome during cell division, thus enhancing vector persistence while avoiding the risks of insertional mutagenesis normally associated with AAV-mediated transduction. The novel expansion of the genetic toolkit with recombinase systems can also improve the process of generating transgenic GECI lines. A recently developed platform combining a Cre recombinase and a transcriptional transactivator (tTA) could greatly enhance the collection of available reporter lines for various GECIs, as tTA-assisted transcriptional amplification can help drive more robust and specific transgene expression in comparison to preexisting Cre systems [149]. Madisen et al. generated a set of Cre/tTA lines which have already validated the insertion of a GCaMP6 cassette at a new targeted docking site on the mouse genome. It is clear that a collection of enhancers, promoter variants, and additional expression elements in animal systems need to undergo rigorous characterization. This is a central tenet to synthetic biology principles and is a vital step to narrow the gap of identification, characterization, and quantification between microbial and animal systems.

### 3.6. Subcellular Localization of GECIs

The flexibility in encoding different peptide motifs into GECIs broadens the various approaches that can be used to target GECI expression to various subcellular compartments in comparison to synthetic calcium dyes. Distinct from cytosolic Ca^2+^ measurements, Ca^2+^ within the ER is often maintained at levels sometimes 5000-fold greater than in the cytosol but can vary due to activity of voltage-gated calcium channels, rate of mitochondrial uptake, or store-operated calcium entry (SOCE) [116]. Significant interest in studying the coordination of Ca^2+^ influx from outside the cell and Ca^2+^ release from the ER has led to multiple developments in specialized indicators [150]. The discovery of the mitochondrial calcium uniporter and its dependence on large, localized [Ca^2+^] has also prompted new research into interrogation of Ca^2+^ homeostasis in the mitochondria and its effect on cell migration and mitochondrial metabolism [151].

The first case of engineering an organelle-targeted GECI by fusing organelle-specific targeting sequences to the indicator utilized an N-terminus mitochondrial targeting sequence with aequorin, but the low photon emission rate of this bioluminescent protein can pose challenges to obtaining subcellular resolution [152]. Since then, multiple GECI variants have addressed the localization of GECIs into the ER, mitochondria, and other subcellular locales. Henderson et al. targeted a low-affinity GCaMP3 variant, GCaMPer, to the ER lumen of human neuroblastoma cells, rat primary cortical neurons, and human cardiomyocytes [116]. A report published around the same time by Suzuki et al. aimed to address the same issue through an alternate adaptation of GGaMP [117]. They engineered a GCaMP2 variant to reduce binding affinity to Ca^2+^ by approximately 1000-fold using site-directed mutagenesis of the CaM domain, validating its ability to report [Ca^2+^]_ER_ dynamics. Calcium-measuring organelle-Entrapped Protein IndicAtor 1 in the ER (CEPIA1*er*) utilizes the well-established ER targeting scheme comprised of an N-terminal signal peptide calreticulin and the C-terminal KDEL retention motif, and this indicator has since been widely cited in multiple studies, including validation in ventricular myocytes [153] and adaptation for a high-throughput screening of drug targets against ryanodine receptors in the ER [154].

Interestingly, researchers found that choice of targeting sequence influences both the overall efficiency of subcellular localization and functional characteristics of the probe, demonstrating that a tandem duplication of a mitochondrial targeting sequence could improve the delivery efficiency of a GECI into the mitochondrial matrix [155]. The approach of choosing the appropriate promoter and tagging sequence for the desired location for GECI expression aligns itself extraordinarily well with the aims of synthetic biology to develop standardized sets of biological parts. Considering the targeting sequences as modular parts, the schemata of modular design are not just limited to the ER but to the tagging of GECIs to other locations with distinctive spatiotemporal Ca^2+^ behavior [156,157]. Replacing the ER targeting sequence of CEPIA3 with a mitochondrial target sequence generated a CEPIA variant which has enabled study of mitochondrial dynamics in diverse cell types [117]. Beyond the signal peptide and terminal motifs used to detect ER or mitochondrial Ca^2+^, another approach incorporated the neuronal hSyn promoter in addition to an N-terminus duplicated mitochondrial targeting sequence, ensuring targeted expression of YC3.6 in specific cell-type mitochondria and demonstrating its in vivo functionality in mice [81]. These researchers did not explicitly approach the construction of their targeted GECIs with a modular, synthetic biology mindset. Nonetheless, these efforts highlight the existing capabilities to develop the necessary genetic parts for GECI expression. The goal is to expand a future standardized collection which has undergone robust in vitro characterization of their function, stability, and behavior. Eventually, this type of tagging can be optimized in more complex genetic circuits that involve multiple localizations of different calcium indicators in order to simultaneously track differences in intracellular Ca^2+^ transients based on different subcellular compartments.

### 3.7. Multiplexing Ca^2+^ Imaging with GECIs

**Figure 3:**
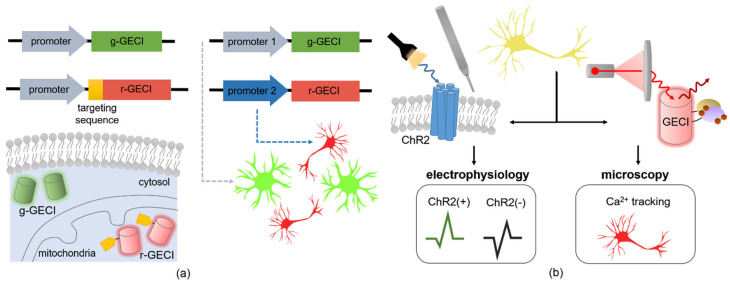
Multiplexing strategies for GECIs. (**a**) Multicolor imaging can be done either within compartments of the same cell type or between multiple cell types in vivo and in vitro. This can be done by fusing a targeting sequence to the N-terminus of a red-shifted variant (r-GECI) and introducing this alongside a standard green-variant (g-GECI) to achieve multiplexed Ca^2+^ tracking in the cytosol and a compartment such as the mitochondria [158]. Another useful application is to utilize cell-type-specific promoters to drive expression of two GECI color variants. This allows simultaneous tracking of Ca^2+^ levels in multiple cell types in vitro or in vivo, such as driving expression of a g-GECI in astrocytes (promoter 1) and an r-GECI in cortical neurons (promoter 2) of the mouse brain. (**b**) A cell expressing both light-sensitive ChR2 and a GECI can enable study of Ca^2+^ dynamics in optically tagged cells [159]. While this optogenetic platform gives an electrophysiological readout of the ChR2-positive neuronal populations in vivo, this can be multiplexed with Ca^2+^ detection. However, since the commonly used GECIs are also blue-light induced, a red-shifted or NIR variant should be used in order to avoid crosstalk with the optogenetic component of the circuit during laser excitation with fluorescent imaging.

Analogous to the discussion of multicolor imaging for synthetic calcium dyes, one drawback of exclusively using GCaMP variants is the limitation of using green fluorescent protein (GFP). This can interfere with cell autofluorescence or optogenetic control [66]. Blue wavelengths of light used to excite green fluorescent GECIs can exhibit excitation overlap with light-sensitive ion channels, allowing undesirable crosstalk with channelrhodopsin-2 (ChR2), a rhodopsin channel [99]. One approach to circumventing this dilemma is by utilizing red GECIs instead of or in addition to the standard green variants. Red-shifted excitation for in vivo imaging can potentially reduce light scattering, and lowered absorption in tissue also reduces phototoxicity. In one early demonstration, Akerboom et al. characterized various mRuby-based GECIs, conducting 2-color Ca^2+^ imaging in the same cell with mitochondria and cytosolic [Ca^2+^] and between different cells in neurons and astrocytes (see Figure 3a) [158].

In one of the more recent red-protein Ca^2+^ detector studies, Dana et al. developed mRuby-based jRCaMP1a and jRCaMP1b and mApple-based jRGECO1a [66]. They found that jRGECO1a exhibited similar performance to the GCaMP6 indicators while jRCaMP1a and jRCaMP1b did not exhibit photoswitching after illumination with blue light in cultured neurons and in vivo mouse, Drosophila, zebrafish and *C. elegans.* Even so, much less research has focused on optimizing SNR of red GECIs versus the GCaMP family, presenting a continued challenge towards keeping up with GCaMP advances. These mRuby- and mApple-based calcium sensors showed improved sensitivity from their predecessors but still exhibited 3- to 4-fold less maximal fluorescence compared to GCaMP6 when bound to Ca^2+^. Currently, simultaneous use of GCaMP6f and jRECO1a still has exhibited overlap, requiring adjustment in order to optimize excitation wavelength [160]. While red GECIs offer a complementary application for use in GFP transgenics which would otherwise interfere with GCaMP, consequent progress must be made to make red GECIs as robust for biological applications.

Far less research is available on NIR fluorescent proteins. The previous studies in this section have dealt almost primarily with visibly fluorescent β-barrel FPs, but the far-red to NIR fluorescent biliverdin-binding FPs have emission peaks in the NIR, from approximately 670 to 720 nm [118]. The latter may offer the ideal tools for increasing the range of colors for multiplexing Ca^2+^ imaging in combination with other optogenetic indicators and actuators. Stamatakis et al. constructed a miniaturized integrated microscope which had two LEDs, one with a 435–460 nm excitation filter for green calcium indicator imaging and another with a 590–650 nm excitation filter for red-shifted opsins [161]. Observing minimal biological and optical crosstalk, this Ca^2+^ imaging allowed observation of behavioral and cellular Ca^2+^ response when optogenetically modulating the basolateral amygdala-to-nucleus accumbens circuit. Adding NIR to the available proteins for recording Ca^2+^ levels will allow even more multiplexing for applications of Ca^2+^ dynamics. Recently, Qian et al. developed NIR-GECO1, using the biliverdin-binding GAF bacterial phytochrome as the fluorescence moiety. Nonetheless, this indicator still exhibited lower brightness, slower kinetics, and less photostability in an inverted response to Ca^2+^ compared to the state-of-the-art green and red-FP derived GECIs [118]. Another research group sought to enhance dynamic range and incorporate a positive rather than inverted response to Ca^2+^ by designing GAF-CaMP2, which combines GAF with a calmodulin/M13-peptide Ca^2+^-binding domain insertion [119]. One drawback was that the addition of a biliverdin chromophore and fusion with sfGFP were necessary for GAF-CaMP2 to function in mammalian cells.

By introducing a number of variants into the repertoire of GECIs with unique spectral properties, synthetic biology applications could address multiplexing by introducing optogenetic capacity to precisely monitor and manipulate neural circuits in order to elucidate brain function. Already, optogenetics has been widely applied to stimulate specific neurons in *C. elegans* [162,163,164], but synthetic biology can combine optogenetics with read-out methods that include phototagging and optotagging to control activity of specific subsets of neurons expressing light-activated ChR2 (see Figure 3b) [100,159,165]. Rather than trying to manipulate the neuronal activity, the intention of this method is to trigger APs when short light pulses are applied to the transduced cells, thus identifying the neuronal population for later monitoring of natural activity [159]. The phototagging of a cell population in a natural environment can supplement study of Ca^2+^ activity using GECIs to simultaneously monitor various neuronal subtypes in vivo. An overview of the optogenetic toolkit pertaining to eukaryotic systems are covered in more depth in the following reviews [135,166]. As researchers develop more optogenetic switches, an ontology-supported online database such as OptoBase offers a powerful tool to scan publications and optogenetic switches available for user application [167].

### 3.8. High-Throughput Methods for Improving GECI Optimization

Traditional imaging technology of large cell populations presents limitations to the capabilities of GECIs to track intracellular Ca^2+^. They are not yet able to address fully the light-scattering properties of the brain that hinder in vivo Ca^2+^ imaging to superficial brain structure [76]. However, the use of miniature, integrated microscopes with microendoscopic lenses has permitted access to deep brain regions (refer to Table 2) and consequent computational techniques to extract Ca^2+^ transient activity from large imaging datasets. Researchers have thus been able to repeatedly visualize large-scale neuronal populations, up to 1000 neurons in one mouse. On the other hand, interest in studying large cell populations in vitro relies on implementing high-throughput assays—that is, parallelized 96-, 384-, or 1536-well formats which can automate >10,000 data points per day—but has been limited to synthetic calcium indicators rather than GECIs [79]. However, Wu et al. directly compared GCaMP6s to Fluo-4 in multiple assays, demonstrating similar indicator results for studying the pharmacology of ion channels and GPCR ligands. By coupling expression of GCaMP6 to a blasticidin resistance gene with a self-cleaving cis-acting hydrolase element, they ensured that progeny of a clonal HEK293-F cell line maintained the stable expression of GCaMP6 necessary for compatibility with high-throughput screening assays. Hence, synthetic biology tools to manipulate levels of gene expression via introducing additional modular genetic parts such as promoters and selection markers can usher in GECIs for use in high-throughput analysis. With comparable measurements of dynamic range, assay reproducibility, and potency measurements of GECIs versus well-characterized synthetic dyes, this once again expands the toolkit available for many high-throughput strategies often applied to modern drug discovery.

The historical approach for optimizing GECIs makes use of high-throughput options that allow screening of recombinant candidate proteins devised by targeted genetic mutations. The most common way of doing so is to develop platforms that facilitate high-throughput screening of a large number of *E. coli* bacterial colonies which express GECI proteins [168,169]. Bacterial colony screening offers a cost- and time-effective way to screen large numbers of biosensor variants which exploit various energy transfer mechanisms [168]. First conducted in bacteria, a majority of the screening for optimal variants have been tailored towards fluorescence imaging performance, but expanding these protein screening platforms can include optimization for absorption-based imaging as well [169]. Screening can also be conducted directly in eukaryotic cells, such as the structure-guided mutagenesis and neuron-based screening for optimizing GCaMP6 with various modes of in vivo imaging [114,127]. In addition, randomized libraries of artificial transcription factors are increasingly beneficial tools for functional genomic studies and potentially for rational engineering of GECIs [170,171]. The traditionally cumbersome requirements for in vitro tissue culture maintenance slow down efforts to explore mammalian tissue engineering. However, the optimization of various cell-free protein synthesis reactions run in microfluidic chambers or microchips may encourage high-throughput prototyping in a mammalian cell-free protein synthesis system [172,173].

While most GECIs utilizes sophisticated imaging techniques (refer to Table 2) for quantitative monitoring of cellular-resolution activity, particularly of transient GECI responses following [Ca^2+^] rises, the development of CaMPARI, or calcium-modulated photoactivatable ratiometric integrator, moves away from online monitoring to a high-temporal-resolution “active snapshot” of large tissue volume [174]. Demonstrated in larval zebrafish and *Drosophila*, as well as in head-fixed mice and adult flies, CaMPARI undergoes an irreversible green-to-red conversion when elevated intracellular Ca^2+^ and experimenter-manipulated illumination occur simultaneously. This example elucidates a promising mechanism for synthetic biology to elaborate upon, as it is well within the current toolkit to develop tunable Ca^2+^ switches or stable integrators of Ca^2+^ which may be implemented in neural and other genetic circuits (see Section 3.5 on optogenetic applications for multiplexing). Overall, these engineered variants will play a crucial role in synthetic biology applications, going beyond multicolor imaging of neural activity to creating programmable transcriptional outputs and triggering key cellular events.

## 4. Calcium-Responsive Nanoparticles

### 4.1. Overview

Nanotechnology finds applications across numerous disciplines due to the stability of various nanoparticles in different conditions and physical properties, offering a flexibility that can be applied to detect Ca^2+^ in extracellular and intracellular capacities. A key feature of in vivo systems is a reliable ability to measure Ca^2+^ dynamics in large volumes using intact tissue. Particularly in the brain, variations in extracellular [Ca^2+^] are essential for proper synaptic activity, and nanoparticles have thus helped interrogate the voltage-induced influx of Ca^2+^ into the cell which reduces extracellular Ca^2+^ from resting levels of approximately 1 mM [175]. Relevant extracellular Ca^2+^ changes can last on the scale of tens of seconds, which can cause a drop in extracellular Ca^2+^ to concentrations as low as 100 µM. Synthetic calcium dyes and GECIs do not have the capability of detecting the relatively higher concentrations in the extracellular Ca^2+^ environment due to their higher binding affinities. While the nanoparticle Ca^2+^ detection field has developed in a niche heavily focused on extracellular Ca^2+^, this section will cover some of these technologies which have potentially useful applications for intracellular Ca^2+^ detection as well.

### 4.2. Enhancing Calcium Dye Functionality and Tunability

Though molecular probes often improve aspects of performance one at a time, whether K_d_, ratiometric capacity, or other functionalities mentioned previously, the nanoparticle matrix has the advantage of being able to incorporate multiple agents for simultaneously tuning. The ion-selective optode design incorporates an ionophore which is a highly selective but optically silent ion carrier, a chromoionophore to serve as a fluorescent reporter, and an ion exchanger to remain electroneutrality [67]. Changing the ratio between the three components modifies the sensing range of the probe. A similar design was also applied by another set of researchers constructing fluorescent calcium-sensitive nanospheres surrounded with lipophilic matrix material to act as a chromoionophore [176]. This enabled intracellular Ca^2+^ imaging in the visible region or NIR region, such that a chromoionophore controlled the fluorescence of the nanosphere. The spectral range was adjustable depending on which chromoionophore was being chosen, and the nanosphere exhibited a rapid response time to Ca^2+^ and 24 h fluorescence.

Calcium-responsive nanoparticles can potentially enhance imaging with preexisting dyes (refer to Table 4). An important development centered on Photonic Explorers for Bioanalysis with Biologically Localized Embedding (PEBBLEs), which incorporate a fluorescent indicator such as Rhod-2 inside a nanoparticle matrix [31]. This can achieve ratiometric measurements even with non-ratiometric probes by incorporating both the sensing indicator and reference dye. The inert polymer matrix can protect the cellular environment from potential cytotoxicity of indicators while protecting the indicator from cell components. The encapsulation of the rhodamine-based fluorescent calcium indicators within the PEBBLE nanosensor provided a nanomolar dynamic sensing range for intracellular Ca^2+^, which can be useful for measuring cytosolic free Ca^2+^. A similar study utilized silicon nanowires (SiNWs) as substrates to anchor small molecules [177]. This configuration of a 1D fluorescence sensor utilized a red-emitting ruthenium-based dye as a reference molecule and green-emitting Fluo-3 as the response molecule to detect Ca^2+^ covalently immobilized on the surface of SiNWs. Therefore, leakage and drift of small molecule or nanoparticle-based fluorescent probes from the cell was minimized, and transfection steps that would be used for GECIs were unnecessary. A micromanipulator successfully located the SiNWs in target regions at the subcellular level, enabling recognition of differences between [Ca^2+^] in the cell body and neurites of neurons.

### 4.3. Magnetic Resonance Imaging of Nanoparticles

Potential avenues of exploration into imaging probes for measuring extracellular Ca^2+^ in vivo pushed researchers to find a molecularly specific analogue to functional magnetic resonance imaging (fMRI) in order to map brain activity. While fluorescent Ca^2+^ sensors have been used to measure extracellular Ca^2+^ levels, these preexisting compounds have binding constants which are too high for the targeted range of 0.1–1.0 mM and are ultimately unsuited for deep-tissue study [183,184,185]. To address this, Okada et al. engineered an improved MaCaReNa probe with synaptotagmin proteins, an endogenous component of synaptic neurotransmitter-release machinery which naturally respond to the narrow extracellular [Ca^2+^] fluctuations [175]. Combining the synaptotagmin 1 domains with lipid-coated iron oxide nanoparticles, MaCaReNas could be detected with MRI and display a Ca^2+^-dependent increase in the strength of a contrast agent. This shows promise as a suitable molecular-imaging paradigm for monitoring Ca^2+^ dynamics in the interstitial space of the brain over a time interval of seconds to hours. Barandov et al. also wanted to capitalize on the benefits of MRI, specifically because it offers extensive penetration depth and field of view [178]. They synthesized a manganese-based paramagnetic contrast agent, ManICS1-AM, which is designed to permeate cells and undergo esterase cleavage similar to other synthetic calcium dyes. Responses were shown to directly parallel signals obtained with fluorescent calcium indicator Fura-2FF-AM. Due to the modularity of this sensor, the membrane-permeable components, the manganese moiety, Ca^2+^-specific chelator, and the linker between them can be modified in order to optimize Ca^2+^ affinity and the paramagnetic complex interaction. This recent study can be considered the first example in which Ca^2+^ fluctuations in an in vivo rat brain were monitored directly at the intracellular level via fMRI.

To improve imaging capabilities, one study built a Ca^2+^-binding nanoprobe which emitted a NIR fluorescence signal [186]. Specifically designed to have high affinity for bone minerals containing calcium phosphates, the self-assembled nanostructures could be utilized to visualize intracellular Ca^2+^ levels in vitro and bone tissues in vivo. Macrophages and osteogenic-macrophages were treated with the nanoprobe, and NIR fluorescence signals coincided with the green fluorescence signals of the calcium indicator Fluo-3 in co-stained cells, suggesting that this nanoprobe has the potential to detect and visualize intracellular Ca^2+^ levels. Going forward with this method will require more validation and comparison of its efficacy to other calcium indicators used to study bone tissues, especially in vivo. Another contrast agent which is bioresponsive to intracellular Ca^2+^ at concentrations between 1 and 10 μM has recently been developed by Adams et al. [179]. The particular use of the conjugated NIR dye leads to a significant increase in uptake of small molecules, coincidentally improving cellular entry of the gadolinium ion (Gd^3+^) complexes used as a contrast agent. In addition to this serendipitous event, the use of an NIR probe also allows in vitro and in vivo optical co-registration of the uptake and subcellular localization of the agent using NIR fluorescence imaging. Taken together, this multimodal MR contrast agent brings the field closer to successful study of intracellular Ca^2+^ flux in vivo that take advantage of the benefits of MRI.

### 4.4. Alternative Optical Techniques to Detect Nanoparticles

Near-field fluorescence (NFF) can improve the fluorescence properties of fluorophores when utilizing metal nanoparticles [4]. When the fluorophore is localized within near-field range of the metal nanoparticle surface, the excitation or emission of the fluorophore can couple with the local electromagnetic field generated by the metal nanoparticles, otherwise called the surface plasmons. In turn, excitation and emission rates are increased significantly while also extending photobleaching time, which is a significant drawback to NIR fluorophores [187]. The shaped metal nanoparticles including metal nanoshells or nanorods, such as gold nanorods (AuNR), can display their surface plasmons at longer wavelengths in the NIR region compared to spherical ones which display plasmons at the visible range [4]. When the imaging contrast agent, composed of an ICG-HSA-Au complex, was injected underneath the skin surface of mice, the luminescent nanoparticles showed 5-fold brighter emission spots compared to conjugates without the AuNRs. Consequently, AuNRs have the potential for detecting voltage-sensitive [Ca^2+^] in cells and living animals with high sensitivity.

As seen in Section 3.3, fluorescence-resonance energy transfer (FRET) is one tool for detecting GECIs but also has more recent nanoparticle applications [181]. Building off the previously mentioned gold nanoparticle technology, researchers have recently employed a semiconductor quantum dot (QD)-gold nanoparticle which can detect Ca^2+^ inside cells upon introduction with a CPP [180]. The DSS peptide which permits successful endocytosis of the DNA aptamer-based nanoparticle has not been previously characterized but may have potential applications for introducing FRET-based Ca^2+^-detecting optical sensors intracellularly, as do other direct delivery methods that involve CPPs such as H11 [181,182]. Overall, calcium-sensitive nanoparticles are a less popular tool and have undergone less characterization for intracellular Ca^2+^ studies. Current research nonetheless shows promising avenues for future studies on tunability and sensitivity to in vivo imaging.

## 5. Novel Calcium Synthetic Biology Approaches

### 5.1. Overview

Synthetic biology focuses on utilizing standardized and interchangeable biological parts with an engineering approach to design devices, systems, and organisms for a useful, novel function. The past decade has brought about significant strides in expanding a synthetic biology toolkit, and one such frontier focuses heavily on controlling multicellular systems and tissue formation. Requiring precision and complexity, this involves either directly or indirectly regulating cellular organization by engineering cellular signaling [188]. Ultimately, these advances can enable synthetic biology inspired therapeutic strategies to remotely control expression of therapeutic genes. Cells are constantly sensing and responding to extracellular stimuli, and synthetic biology can take advantage of varied input signals, including inducer molecules, pH, and light, to carry out a desired cell response [135]. Expanding the study of calcium signaling to include more indirect methods has been demonstrated by Furlan et al.’s study of SOCE as a proxy of Ca^2+^ transients [189]. As synthetic biology grows in use, we will discuss the prospect of tools that focus on interrogating Ca^2+^ via indirect measurements or engineering novel calcium-dependent transcription factors and responsive elements.

### 5.2. Calcium-Dependent Transcription Factors

In recent years, a number of different research groups have constructed engineered calcium-responsive transcription factors that focus on detecting or triggering transient Ca^2+^ influx. Calcium-dependent transcription factors are particularly attractive as synthetic biology tools because of the numerous physical and chemical stimuli which can induce the cellular uptake of Ca^2+^, such as the activation of GPCRs and Ca^2+^-selective ion channels [190]. An artificial Ca^2+^-dependent transcription factor in baby hamster (BHK) cells was able to detect sustained intracellular Ca^2+^ elevation [191]. An increase in intracellular Ca^2+^ level induces activation of calpain, which cleaves a calpain-sensitive sequence of the Ca^2+^ transcription factor in the cytosol. This modified transcription factor can bind a TetO element and induce expression of a reporter luciferase. Such a study provides a proof of concept that Ca^2+^ input signals can be decoded to produce a desired output, such as bioluminescence output via luciferase. Another study had the goal of developing a calcium-dependent transcription factor, this time reporting an NFAT-based genetic circuit that regulates transcription in response to external physical or chemical signals [192]. The signals trigger increases in cytosolic [Ca^2+^] while limiting undesired off-target activity by eliminating endogenous NFAT site binding, thus ensuring orthogonality. Ultimately, these various synthetic transcription factors which are activated by physiological Ca^2+^ can serve as an interface for endogenous cell systems and synthetic systems.

A significant advance in the realm of stable Ca^2+^ integrators in eukaryotes is the use of Tobacco Etch Virus (TEV) proteases, having previously been deployed in neurons [193]. While classic approaches take advantage of immediate early gene upregulation in response to neural activity for marking active populations of neurons, this suffers from inconsistent upregulation in different neurons [193,194]. The Split TEV protease, Ca^2+^ Activated Neuron Recorder (SCANR), is a modular Ca^2+^ reporter system which remains catalytically incompetent until a Ca^2+^ spike triggers reconstitution and activate transgenes of interest [193]. This enzyme reports artificially applied Ca^2+^ spikes in HEK293 cells or in neuronal signaling in cultured rat hippocampal neurons. SCANR’s modularity offers important flexibility for future synthetic biology research on incorporating optogenetic modules for spatial and temporal control of the enzyme’s activity.

Wang et al. wanted to selectively characterize and manipulate neurons by engineering a different transcription factor system, Fast Light- and Activity-Regulated Expression (FLARE) [194]. Ca^2+^-mediated induced proximity of active calmodulin and an M13 peptide leads to TEV protease cleavage and release of a membrane-constrained transcription factor, which drives the expression of opsins and other genetically encoded tools when it senses coinciding elevated cytosolic Ca^2+^ levels and externally applied blue light. With modular design and minute-scale temporal resolution, FLARE offers an important system in neuroscience to measure responses to inputs as varied as calcium channels and engineered ligand-receptor pairs, finding numerous approaches that extend even beyond Ca^2+^ to result in transcription factor release [195]. These advances can be accelerated with implementation of synthetic biology strategies for directed evolution of proteins (see review by Kang et al. [196]), which must involve detailed biochemical and biophysical characterization complemented by computational strategies to reduce the costs of empirical optimization [197].

### 5.3. Modification of Ca^2+^-Related Cis-Elements

One powerful tool to alter genomic DNA or the epigenome is the CRISPR/Cas9 system [198]. Programmable sequence-specific endonucleases can allow precise editing of loci to interrogate, silence, remove, add, and modulate genetic elements. Combining CRISPR with optogenetics uses light to provide an external locus and timing of control for genomic editing while modifying Cas-9-mediated transcriptional activity. Nguyen et al. constructed a genetically encoded photoactivatable Ca^2+^ releaser under the control of Opto-CRAC, a light-inducible Ca^2+^ release activated channel (CRAC) [199]. Ca^2+^ influx as a result of Opto-CRAC activates calcineurin, which dephosphorylates NFAT and consequently results in its nuclear translocation. Specifically, the OptoCRAC channel remotely controlled the Ca^2+^ signal so that an NFAT fragment fused with dCas9 and a VP64 transcription factor would translocate into the nucleus to control gene expression. In Xie et al.’s experiment, a synthetic promoter P_NFAT2_ containing NFAT repeats from murine IL-4 promoter was most responsive to chemically induced membrane depolarization by KCl [200]. By simultaneously transfecting with voltage-gated Ca_V_1.2 channels, these channels fine-tuned calcium signaling so that cells exhibited amplified excitation-transcription coupling and higher sensitivity [200,201]. In using NFAT as an actuator for decoding calcium signaling, it is important to recognize that various Ca^2+^ dependencies of NFAT isoforms permit agonists to recruit different combinations of isoforms as stimulus strength increases [202]. This means that cytoplasmic transcription factor NFAT isoforms are differentially activated by specific subcellular calcium signaling patterns.

Beyond in vitro systems, Tastanova et al. demonstrated a working hypercalcemia biosensor in both HEK293 cells and in vivo mice [203]. The Ca^2+^-sensing receptor (CaSR), a GPCR that normally manages Ca^2+^ homeostasis in humans, was instead ectopically expressed in mice. The researchers constructed a CaSR-dependent calcium-sensitive promoter (P_Ca4_) containing three SRE and six NFAT operator modules 5′ of a minimal version of the constitutive human CMV immediate early promoter. Under the control of this promoter, HEK293 cells produced melanin in response to sustained Ca^2+^ sensed in blood, which was also validated in wild-type mice implanted with engineered cells. Such cell-based biosensors can provide cheap, portable, and simple methods of detecting molecules of interest, but a review by Hicks et al. discusses the lack of reliability and long-term stability of biosensors so far as barriers to scaling up applications [204].

**Figure 4:**
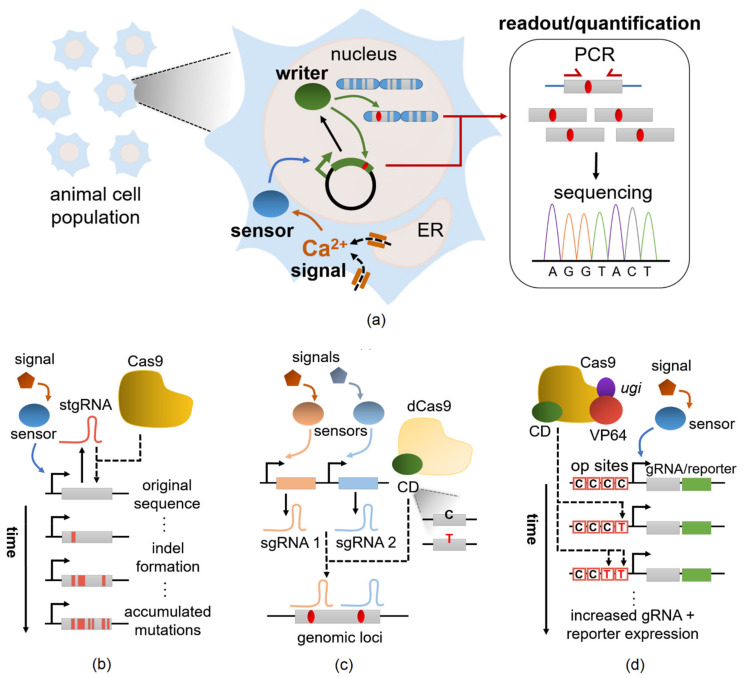
Conceptual representation of cellular recording devices. (**a**) When engineered into animal systems, base-editing systems can sense a signal, in this case the detection of cytosolic Ca^2+^, by incorporating a read-write system. The writer is introduced by a plasmid and once translated, will base edit at genomic or plasmid loci. These loci can consequently be read and quantified by high-throughput sequencing (HTS) methods. (**b**) Mammalian Synthetic Cellular Recorders Integrating Biological Events (mSCRIBE) [205]. mSCRIBE uses a self-targeting guide RNA (stgRNA) that directs the Cas9 writer to a DNA locus which accumulates mutations over time. The initial stgRNA is transcribed from a plasmid, and multiple rounds of transcription and DNA cleavage progressively mutate the original stgRNA region so that it can eventually be sequenced to act as a cellular record. (**c**) CRISPR-mediated analog multi-event recording apparatus (CAMERA) [206]. CAMERA leverages a combination of multiple single-guide RNAs (sgRNAs) to target different genomic loci along with a writer (the CD base-editor fused to a dCas9) to record nucleotide changes from C•G → T•A. The percentage of nucleotide changes at the specified loci can be quantified with a sequencer. (**d**) DNA-based Ordered Memory and Iteration Network Operator (DOMINO) [207]. This platform leverages a writer that consists of a Cas9 nickase fused to CD and transcriptional activator VP64. A uracil DNA glycosylase inhibitor (ugi) blocks cellular repair machinery to improve DNA writing efficiency. Incoming guide RNAs (gRNAs) target the Cas9 complex to the upstream operator (op) sites, resulting in base-editing mutations on the gRNA sequence that generate memory states readable by HTS. VP64 adds another method for quantifying the loci by also converting the signal into transcriptional output of a reporter that can be measured by a functional assay.

While light-controlled activation of various signaling pathways has already been achieved, utilizing NFAT or cAMP response element (CRE) to name a few Ca^2+^-driven pathways, this raises the important possibility of expanding the array of options for understanding endogenous cell responses to Ca^2+^. A majority of the calcium indicators discussed so far rely on fluorescent imaging, which come with phototoxicity and photobleaching effects discussed in previous sections. While longer wavelength or bioluminescent probes can ameliorate some of these issues, a newer foray of synthetic biology into DNA-based cellular recorders may offer an opportunity to preclude a fluorescent imaging requirement altogether. Of the hundreds of readouts for calcium signaling broadly available, these synthetic biology-based recording devices specifically have the potential to capture stimulus data from whole animal cell populations by using a DNA-based output detectable with HTS techniques (see Figure 4a), obviating the need for fluorescent readout. However, there are currently a limited array of methods that have been refined for continuous DNA-based cellular recording in eukaryotic cells, including mSCRIBE [205], CAMERA [206], and DOMINO [207] (see Figure 4b–d). When linking to stimuli such as Ca^2+^ flux or associated downstream targets of Ca^2+^, these DNA modifications should theoretically represent cellular histories which can be read even after cell death [206]. The DNA readout offers both duration and intensity information about a stimulus in animal cell populations. This population-wide metric could eventually be optimized for cellular recording of Ca^2+^ signals in regions particularly difficult to access in real time using current imaging techniques, such as in utero, in thick brain tissue over long distances, or during animal behaviors sensitive to concurrent imaging [193,207].

### 5.4. Synthetic Biology Workflow Optimization for Calcium Biosensors

Just as GECIs and synthetic dyes have undergone their own optimization, cell-based biosensor optimization via synthetic biology needs to similarly apply rational methods. More strategies that can expand the range of de novo allosteric transcription factors for Ca^2+^ detection will be vital to biosensor optimization [208]. In particular, the use of directed evolution to change specificity patterns can enhance Ca^2+^ detection by introducing intracellular synthetic circuits not limited to natural sources of Ca^2+^-sensing machinery. Directed evolution has existed for decades in protein engineering, but Saito et al. recently used advances in machine learning to develop an artificial intelligence-guided mutagenesis platform that could alter the properties of GFP [209]. With two rounds of protein-variant libraries, they were able to discover multiple mutants with longer wavelengths than a yellow fluorescent protein. This computational guidance is a promising avenue for moving forward directed evolution approaches.

Information derived from mathematical models can point to necessary modifications that need to occur with various genetic parts. These performance models should account for concentration levels, biosensor kinetics based on enzymes involved in each part of the system, and alteration of copy number of promoters to improve sensitivity [210]. When developing alternative paradigms, such as relying on expression of recombinant synthetic proteins fused to enzyme fragments, that no longer rely on transcription-based circuits, mathematical models must build in new logic functionality [211,212]. For example, an optimization framework for modeling a protease-based circuit that includes the kinetics of processes such as protein degradation in cells can be adapted for future models in animal cells, often exceeding the complexity of the biomolecular logic gates devised for current circuits and networks [212]. Optimization of cell-based biosensors have previously been undertaken in ad hoc fashion by altering various parts of the genetic circuit and observing the consequent effect, but mathematical modeling can target which part—dynamic range, limit of detection, or the operating range—of the response curve to address [204,213]. Ultimately, it will be necessary to conduct mathematical modelling of the role of individual sensor components to create a response curve [213], determining the limit of detection by altering transcription factor concentrations [214], and modifying promoters to modulate the dynamic range and SNR of the sensor [135,203].

## 6. Conclusions

Calcium signaling dynamics continue to have key implications across all reaches of biological applications, from neuronal communication to T-cell activation to muscle bioenergetics. It is therefore essential to evaluate the best possible detection methods for Ca^2+^ and the drawbacks or confounding factors that can influence data collection. We have an expansive body of research on Ca^2+^ detection methods, from synthetic calcium dyes to GECIs, which has enabled significant optimization of these tools. However, even within recent years, more research has revealed that introducing these calcium sensors into cells has significant effects that are not orthogonal to endogenous systems. These include potent suppression of Na^+^- and K^+^-dependent adenosine triphosphatase activity by chemical calcium indicators [215], interruption of L-type voltage-gated calcium channels by GCaMP6 [115], or otherwise alteration of the property of Ca^2+^ signals [1,89].

When considering the development of successful probes, researchers should consider Ca^2+^-binding properties, capacity to control localization in desired subcellular compartments, fluorescence lifetime, and stability to photobleaching. Of course, these concerns naturally arise due to the fact that the current, popular methods of detecting Ca^2+^ (e.g., Fluorescein and Rhodamine dye families or single protein-based GECIs) all rely on a fluorescent readout. The resultant phototoxicity and inability to capture deep tissue all present limitations to biological applications, particularly in vivo [175,183,184,185]. Bioluminescent GECIs or NIR dyes are some more recently introduced methods which can circumvent some of these issues, but these Ca^2+^ sensors will need further modification and optimization in order to represent the same dynamic range and SNR exhibited by the most recent iterations of GCaMP6s or dyes. Future research on current Ca^2+^-binding proteins and dyes should focus on multicolor calcium sensors which achieve fast kinetic responses and Ca^2+^ affinity optimized for a range that encompasses the various concentrations of Ca^2+^ in cellular components and the cytosol [216]. Synthetic biology approaches towards biosensors necessarily focus on dynamic range, sensitivity, selectivity, and orthogonality [217], and numerous approaches including site-directed mutagenesis, directed evolution, rational design, and expression vector engineering have already guided us towards this path of optimization regarding calcium signaling research in recent decades.

The advantage of future applications of synthetic biology lies in a combinatorial approach, which synthesizes these desired features for Ca^2+^ detection and provides a potential toolkit to do so. Moving in the direction of synthetic biology, a cytosolic Ca^2+^ sensor would take advantage of endogenous Ca^2+^-binding properties while removing the limitations presented by the more popular fluorescent methods. Although more work has been done regarding the design principles of transcription-factor-based biosensors in prokaryotic systems, the same issues must be tackled in order to have the desired precision and range in eukaryotic systems. As synthetic biology tools become increasingly common at the intersection of biology, chemistry, and physics, novel classes of detection methods for Ca^2+^ are ripe with research possibilities. We should wholly embrace the diverse and versatile array of experimental methods utilizing synthetic biology that continue to be refined and expanded, and the concomitant ability to achieve more precise spatiotemporal measurement of Ca^2+^ dynamics will enhance the capacity to analyze the diverse effects of Ca^2+^ on biological systems in basic science and medical applications.

## Figures and Tables

**Table 1:** Properties of commonly used synthetic calcium dyes.

Synthetic Dye	Class	Excitation/ Emission (nm)	Kd for Ca^2+^ (μM)	Time to Peak (ms)	koff (s^−1^)	Dynamic Range	References
Fura-2	ratiometric, dual excitation	363, 335/512	0.23	NR	23	45.7	[16,21,22]
Mag-Fura-2	ratiometric, dual excitation	329, 369/511	25 (1.9 mM for Mg^2+^) *	5	>5000	0.13 for 1 AP	[19,22]
Indo-1	ratiometric, dual emission	331/485, 510	0.36	14	52	12.9	[16,21,22]
OGB-1	single wavelength	488/515	0.17	53	73	>5.7	[23,24]
Cal-520	single wavelength	492/514	0.32	40	NR	>0.6 (puff)	[23,24]
Fluo-4	single wavelength	494/516	0.35	>40	0.021	∼100	[24,25]
Mag-Fluo-4	single wavelength	485/520	22 (4.7 mM for Mg^2+^)	5.3	NR	1.61 (1 AP)	[18,26]
Fluo-3	single wavelength	506/526	0.39	NR	143	50–200	[25,27]
CaRuby-Nano	single wavelength	575/605	0.26	0.55	NR	50	[28]
CaSiR-1	single wavelength	650/654	0.58	NR	NR	5–20 (1–3 APs)	[29]
Ca-NIR	ratiometric, dual excitation	616, 666/684	∼8	NR	NR	NR	[30]

NR—not reported. *Indicators generally have more than 10^5^-fold higher selectivity for Ca^2+^ over Mg^2+^. Otherwise, K_d_ for Mg^2+^ is also listed. Intracellular concentrations of free divalent cations, such as Mn^2+^, Zn^2+^, or Cd^2+^, are much lower than their affinities to BAPTA, leading to little interference in an intracellular milieu for Ca^2+^ sensing with BAPTA-type indicators [31].

**Table 2:** Applications for various imaging techniques to capture Ca^2+^ levels across animal cell types.

Imaging Technique	Representative Targets	Frame Rate	Indicator Types *
Two-photon microscopy	in vitro: cortical; hippocampal; immortal lines (e.g., HeLa); lymphocyte	6–500 Hz	Fluo-4, Rhod-2, OGB-1, Cal-520 [23,37] GCaMP-X, Twitch-1/2B, jRGECO1a [65,66] Nanosensors [67]
	ex vivo: 300 μm brain slice; epithelia	700 Hz	OGB-1, Cal-520 [23] GCaMP3 [68]
	in vivo (up to 600 μm depth): lymph nodes; pancreas; whole brain; whole-mount embryo	<1 Hz–700 Hz	Fura-2, Fluo-4, OGB-1, Cal-590 [34,69] GCaMP5G/6, Twitch-1, Salsa6f, YC-Nano15 [65,70,71,72,73,74]
	in vivo: deep neuronal tissue in awake animal (using microendoscopy)	Commonly 30 Hz, up to 10 kHz [75]	See discussion of method for various dyes [34,69] and GECIs [76,77]
Confocal (single-photon) microscopy	in vitro: adult stem; astrocyte; embryonic; myocyte; hippocampal; cortical; immortal lines (e.g., PC-3); lymphocyte	0.1–20 Hz	CaSiR-1, Fluo-4, Fura-2 [3,29,41,78,79] GCaMP3/6, Salsa6f, YC2.6 [72,80,81] PEBBLE [31]
	ex vivo: aortic rings; brain slices; bone; Drosophila larval wing discs; Xenopus ectoderm (animal cap); pancreatic islet	0.1–10 Hz	Fluo-4, Fluo-8, OGB-1 [82,83,84] GCaMP5G/6, YC-Nano3GS [73,78,85]
	in vivo (up to 370 μm depth): whole brain	6 Hz, up to 70 Hz	GCaMP6 [86]
Non-confocal light microscopy	in vitro: motor neuron	25 Hz	GCaMP6 [87]
Single-lens digital	in vitro: immortal lines (e.g., HEK293); cortical	Up to 30 Hz	Fluo-4 [88]
Plate reader assays	in vitro: myocyte	16–60 Hz	Cal-520, Fluo-4 [79,89] GCaMP6 [79]
Flow cytometry	in vitro: lymphocyte; platelets	N/A	Fluo-4, Fura-2, Indo-1 [41,90,91]

* Citations are not comprehensive, some representative studies are listed.

**Table 3:** Properties of the most commonly used GECIs.

GECI	Class/Color	Excitation/ Emission (nm)	K_d_ for Ca^2+^ (μM)	Half Decay Time (s) for 1 AP	SNR	Dynamic Range	References
GCaMP6s	single-FP, GFP	485/510	0.144	0.55	4.4–66 (1–10 APs)	63.2	[24,98,114]
GCaMP6m	single-FP, GFP	485/510	0.167	0.27	20 (40 Hz)	38.1	[98]
GCaMP6f	single-FP, GFP	485/510	0.375	0.11	3.2–50.9 (1–10 APs)	51.8	[24,66,98,114]
jGCaMP7f	single-FP, GFP	485/510	0.174	0.265	17.2	40.4	[114]
GCaMP-X	single-FP, GFP	488/510	2–40	0.60	~18	5	[115]
GCaMPer	single-FP, GFP	490/540	~400	NR	NR	14	[116]
R-CEPIA1*er*	single-FP, RFP	498/560–800	565	NR	NR	15.6	[117]
jRGECO1a	single-FP, RFP	570/600	0.148	0.39	NR	11.6	[66]
NIR-GECO1	single-FP, GAF (NIR)	678/704	0.215	1.93	0.52	8	[118]
GAF-CaMP2	single-FP, GAF (NIR)	642/674	0.466	NR	NR	1.69	[119]
Twitch-1	FRET, YFP/CFP	432/450–600	0.25	0.80	NR	4	[92]
Twitch-2B	FRET, YFP/CFP	432/450–600	0.20	2.80	10–15	8 (1–AP)	[92]
TN-XXL	FRET, YFP/CFP	440/470	1.03	0.88	NR	1.6–11.2 (2–20 APs)	[92,120]
YC3.6	FRET, YFP/CFP	440/535	0.25	0.34	0.73	6.6	[121,122]
YC-Nano15	FRET, YFP/CFP	458/535	0.015	3–4	2.9–11.4 (1–10 APs)	14.5	[123]
LUCI-GECO1	Bioluminescent, NanoLuc	––/460	0.283	1.21	NR	26	[99]
GLICO	Bimodal, NanoLuc/GFP	480/520	0.59	2.48	0.4	22	[101]

NR—not reported.

**Table 4:** Properties of calcium-responsive nanoparticles.

Nanoparticle	Fluorophore (Excitation/Emission) (nm)	Range of Ca^2+^ Detection (μM)	Delivery Method	Ratio- Metric?	Response Time	Selectivity	Reference
calcium-optode nanosensor (opCaNS)	CHIII (639 /670) R18 (555/575)	0.038–0.60	microinjection	Y	NR	10^4^-fold over Mg^2+^	[67]
calcium-selective nanospheres	CBDP (480/510) ETH 5350 (645/669)	67	non-specific endocytosis	N	1 s	Against Mg^2+^, K^+^, Na^+^	[176]
SiNWs	Fluo-3 (488/NR)	0.5–1	micropipette via micro- operation system	Y	NR	Against Mg^2+^, Zn^2+^, K^+^, Na^+^	[177]
PEBBLEs	Rhod-2 (540/560–600)	0.293	non-specific endocytosis	Y	2–2.5 s	NR	[31]
magnetic calcium-responsive nanoparticles (MaCaReNas)	N/A	100–1000	intracranial injection into rat striatum	N	4–4.9 s	Against Mg^2+^	[175]
manganese-based intracellular calcium sensor (ManICS1-AM)	N/A	0.20	cell incubation	N	NR	NR	[178]
Ca(II)-responsive NIR multimodal MR contrast	IR-783 (745/810)	1–10	cell incubation	N	NR	10^5^-fold over Mg^2+^	[179]
indocyanine green-human serum albumin-Au (ICG–HSA-Au)	ICG (760/819)	NR	cell incubation	N	0.4–2.3 ns	NR	[4]
DNA aptamer-based optical sensors	QD (375/655)	3.77 × 10^−6^ –0.035	DSS peptide	Y	5–15 min	Against Mg^2+^, K^+^, Na^+^	[180]
QD-CaRuby-CPP	Ca-Ruby (407–545/500–700)	3–20	H11 cell–penetrating peptide (CPP)	Y	k_on_ = 10^8^ M^−1^ s^−1^ k_off_ = 150 s^−1^	NR	[181,182]

NR—not reported.
